# Association between the Composite Dietary Antioxidant Index and severe headache or migraine: results from the National Health and Nutrition Examination Survey

**DOI:** 10.3389/fneur.2024.1407243

**Published:** 2024-08-01

**Authors:** Zhiru Zhang, Xushan Chen, Haotao Fang, Jiechao Ye, Xiaona Tang, Rucheng Huang

**Affiliations:** ^1^Seventh Clinical Medical College, Guangzhou University of Chinese Medicine, Shenzhen, China; ^2^Nursing Department, Baoan District Hospital of Traditional Chinese Medicine, Shenzhen, China; ^3^Department of Encephalopathy, Baoan District Hospital of Traditional Chinese Medicine, Shenzhen, China

**Keywords:** Composite Dietary Antioxidant Index (CDAI), migraine, severe headache, oxidative stress, cross-sectional study

## Abstract

**Background:**

Severe headache or migraine is a neurological disease that seriously affects the quality of human life. Oxidative stress is considered a main factor in the pathogenesis of severe headache or migraine. The Composite Dietary Antioxidant Index (CDAI) is a score calculated using six dietary antioxidant components (including vitamins A, C, E, selenium, zinc, and carotenoid), which represents a person’s level of dietary antioxidant ingredients. Based on the theory of oxidative stress, we speculated that CDAIs may be relevant to the risk of severe headache or migraine, as the relationship between the CDAI and severe headache or migraine is unclear. Hence, the purpose of this study was to explore the relationship between the CDAI and severe headache or migraine in participants.

**Methods:**

We performed a cross-sectional study using data from the National Health and Nutrition Examination Survey (NHANES) that were collected from 2001 to 2004. A total of 4,943 participants were included, of whom 1,232 experienced severe headaches or migraines. Participants’ CDAIs were calculated based on their intake of six dietary antioxidants. We used logistic regression models, limited cubic spline analysis, and subgroup analysis to assess the association of CDAI with severe headache or migraine.

**Results:**

The multivariate logistic regression model (correcting for all potential covariates) revealed that the odds ratio (95% Confidence Interval [CI]) for the association between CDAI and severe headache or migraine was 0.97 (95% CI = 0.95–1.00, *p* = 0.048). Compared with individuals with low CDAIs in Quartile (Q)1, the adjusted Odds Ratio between the CDAI and severe headache or migraine in Q2, Q3, and Q4 were 0.84 (95% CI = 0.69–1.01, *p* = 0.07), 0.77 (95% CI = 0.63–0.96, *p* = 0.017), and 0.73 (95% CI = 0.56–0.95, *p* = 0.02), respectively. Restricted cubic spline regression analysis showed an L-shaped relationship between the CDAI and severe headache or migraine.

**Conclusion:**

Our findings indicate that higher CDAI was associated with a lower risk of severe headache or migraine.

## 1 Introduction

Migraine is a widespread neurological disorder that affects more than 1 billion people worldwide (1), the majority of whom are women. The main manifestation is a severe throbbing headache on one or both sides of the head, mostly occurring on one side. Migraine is often accompanied by nausea, vomiting, and high sensitivity to light and sound, and migraine attacks have a negative impact on the individual’s quality of life (2). Studies have shown that patients with migraine have a significantly increased risk of panic attacks and anxiety (3, 4). Migraine has a high incidence and a long duration, ranking secondn the burden of neurological diseases. The World Health Organization ranks migraine as the third most common disease worldwide and the second most disabling neurological disease. According to the American Migraine Prevalence and Prevention Study, most people with “severe headaches” meet the diagnostic criteria for migraine (5). The pathogenesis of severe headache or migraine is currently uncertain, but oxidative stress is considered the main factor in the pathological process (6–8). Migraine attacks are an adaptive response to a lack of brain energy and elevated levels of oxidative stress (9). Many studies support the hypothesis that oxidative stress is caused by defects in the oxidative energy metabolism of brain mitochondria (10). Oxidative stress refers to an excessive production of highly active molecules, such as reactive oxygen species and reactive nitrogen in the body, which causes an imbalance of the body’s oxidation and antioxidant systems, leading to tissue damage.

Based on the theory of oxidative stress damage, many researchers have confirmed that a single dietary antioxidant component has a beneficial effect on severe headache or migraine. However, the diets of humans contain a variety of nutrients, and the impact on the human body is caused by the joint action of the overall nutritional components. We believe that it is necessary to conduct a comprehensive evaluation of various antioxidant components to explore a meaningful scoring system. The Composite Dietary Antioxidant Index (CDAI), developed by Wright et al. (11), is a comprehensive score that measures a variety of dietary antioxidants, including selenium, zinc, carotenoid, and vitamins A, C, and E. The CDAI is a reliable nutritional indicator that reflects the antioxidant properties of an individual’s diet. Previous studies (11–18) have reported that a high CDAI was associated with a reduced risk of several types of cancer and chronic diseases, the handgrip strength of a group with a high CDAI was higher, and high CDAIs have been confirmed to have anti-aging and anti-depression properties. Furthermore, a high CDAI is related to a lower risk of all-cause mortality and cardiovascular deaths (19).

The results of previous research support the beneficial effect of a higher CDAI in preventing the occurrence and development of a variety of diseases due to antioxidant stress. Oxidative stress is regarded as one of the mechanisms of severe headache or migraine; therefore, we hypothesized that a high CDAI would be related to a lower risk of severe headache or migraine. This study used survey data from the National Health and Nutrition Examination Survey (NHANES) database to conduct a cross-sectional study to investigate the potential relationship between the CDAI and severe headache or migraine.

## 2 Materials and methods

### 2.1 Data source

The NHANES is a research program designed to assess the health and nutrition status of adults and children in the United States (US).^1^ The NHANES included demographic, screening, dietary, laboratory, and questionnaire data collected by trained researchers via home visits, medical examinations, and laboratory tests. The studies involving humans were reviewed and approved by the National Center for Health Statistics Ethics Review Board. Participants provided written informed consent for participation in NHANES. The data were de-identified and all participant data were obtained from publicly available NHANES. Our study is a secondary analysis of the 2001–2004 NHANES data collected by the Centers for Disease Control and Prevention and the secondary analysis does not require additional institutional review board approval.

### 2.2 Screening of the research data

**Figure 1:**
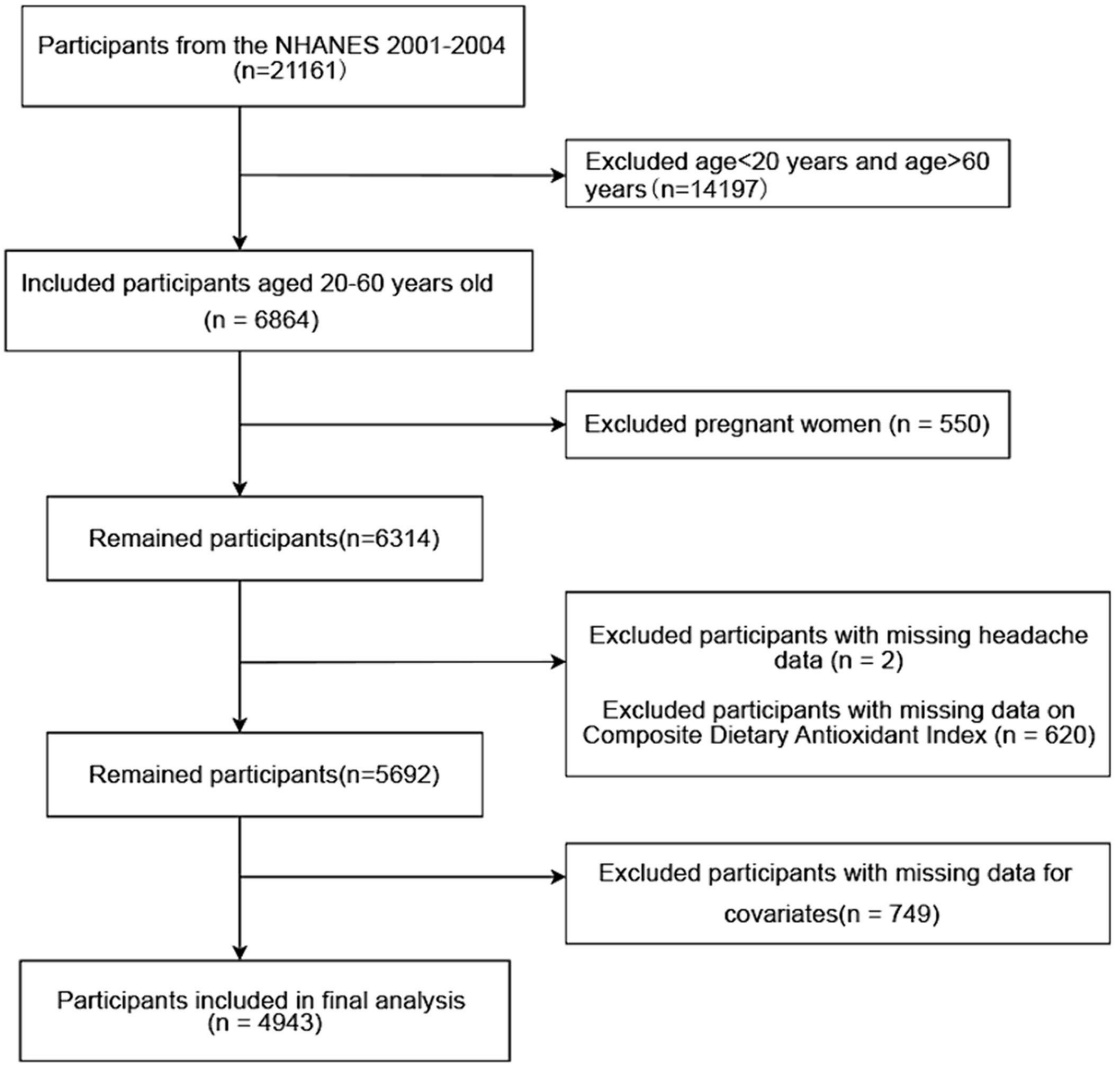
Flow chart of the screening of the subjects. A total of 6,864 participants aged 20–60 years from NHANES 2001 to 2004 were included. Pregnant women and those who miss headache data, diet data, and covariates were excluded. A total of 4,943 participants were included. NHANES, National Health and Nutrition Examination Survey.

This study used the 2001 to 2004 data from the NHANES that were collected from 31,126 participants who respondents to the survey. We excluded 14,197 participants who were younger than 20 years of age and participants older than 60 years. Among the remaining 6,864 individuals aged 20 to 60 years, we excluded 550 pregnant women, as well as individuals who lacked data on severe headache, migraine, diet, or covariates. In the end, a total of 4,943 participants were enrolled in the study (Figure 1). Of these, 1,232 experienced severe headaches or migraines.

### 2.3 Study variables

#### 2.3.1 Exposure variable: CDAI

Information about each of the participant’s food and nutrient intake was collected during the dietary interview component called, “What we eat in America.” Participants were asked to recall specific foods and beverages consumed during the 24 h prior to the interview. As part of the dietary survey, the US Department of Agriculture and US Department of Health and Human Services worked together to determine participants’ nutrient intake. Two days of intake data were recorded for each participant in the 2003–2004 cycle. The first day is collected in the Mobile Examination Center (MEC) and the second day is collected by telephone 3 to 10 days later. In order to be consistent with the data for the 2001–2002 cycle, we used the first day intake data collected in MEC. We used six dietary antioxidant intakes of concern from the NHANES diet interview section: zinc, selenium, carotenoid, vitamin A, vitamin C, and vitamin E. The carotenoid intake was estimated based on the intake of alpha-carotene, beta-carotene, beta-cryptoxanthin, lycopene, lutein and zeaxanthin. In this study, the calculations of dietary nutrient intake did not include nutrients from medications, common drinking water, or dietary supplements.

We used a modified version of the CDAI formula developed by Wright et al. (11, 20, 21) to calculate participants’ CDAIs. Briefly, we normalized the intake of each antioxidant by subtracting the mean and dividing it by the standard deviation; then we added the standardized intake of 6 dietary antioxidants to calculate the CDAI.


$$\mathrm{CDAI}=\overset{n=6}{\underset{i=1}{\sum }}\frac{\mathrm{Individual}\;\mathrm{intake}-\mathrm{Mean}}{\mathrm{Standard}\;\mathrm{deviation}}$$

#### 2.3.2 Outcome variable: severe headache or migraine

Headache diagnoses and assessments were based on participants’ self-reports. Relevant headache information was obtained from the “Miscellaneous pain” section of the NHANES questionnaire. In this section, the specific question was “Have you had severe headaches or migraines in the past 3 months?” If participants answered “Yes,” they were considered to be suffering from severe headaches or migraines. The American Migraine Prevalence and Prevention (AMPP) study (5) showed that 17.4% of individuals reported “severe headache,” of which 11.8% met the International Headache Disorder Type II (ICHDII) criteria for migraine, 4.6% met the criteria for “possible migraine,” and only 1% were identified as “other severe headache.” Given the demographic resemblance of our study’s participants to population samples from epidemiological surveys, it seems reasonable to deduce that most of the participants with severe headaches did indeed have migraines. Therefore, it is justified to label participants who reported severe headaches or migraines in the NHANES as migraine sufferers. Previous studies using NHANES data to examine the relationship between migraine and nutrients (22, 23), body mass index (24), and serum markers (25) have also used the same inference to assess migraine.

#### 2.3.3 Baseline data collection

Based on the available literature, we evaluated the potential confounding factors that were likely to affect the link between the CDAI and severe headache or migraine (sex, age, poverty income ratio [PIR], marital status, educational level, body mass index [BMI], smoking, energy, protein, carbohydrates, C-reactive protein, total cholesterol, and chronic disease status, such as stroke, coronary heart disease, hypertension, diabetes, and asthma).

We divided marital status into unmarried, divorced/widowed/separated, and married/cohabiting. Educational level was classified as: less than high school, high school or equivalent, and more than high school. The PIR was calculated by dividing a household’s total income by the federal poverty line for that year. Participants’ BMI was defined as the ratio of weight (kg) to height squared (m^2^). Smoking status was classified as never smoked/former smoker and smoking/current smoker. Protein-, carbohydrate- and energy intake were determined using the total dietary nutrient intake questionnaire. The diagnoses of stroke, coronary heart disease, and asthma were determined, based on self-reported results from the Medical Conditions section of the questionnaire. The diagnoses of hypertension and diabetes were determined in accordance with BPQ020 and DIQ010, respectively, in the questionnaire.

### 2.4 Statistical analyses

IBM SPSS Statistics V26 software and Free Statistics software were used to organize and analyze the data. *p* < 0.05 was considered statistically significant. The CDAIs were converted to a quartile array to describe and analyze participants’ demographic characteristics. Categorical variables are expressed as proportions (%) and continuous variables are described as either mean [standard deviation, (SD)] or median [interquartile range, (IQR)]. Differences were analyzed between the CDAI quartile arrays using the Chi-square, Variance analysis or Kruskal-Wallis H test. We used logistic univariate analysis to explore the relationship between the covariates we identified and severe headache or migraine.

We used a logistic regression model to investigate the association between the CDAI, CDAI category, and severe headache or migraine. Model 1 had no covariate adjustments. According to the relevant epidemiological research literature, age and sex are closely related to the risk of severe headache or migraine (26, 27); therefore, sex and age were adjusted in the multivariate analysis (Model 2). Reports in the existing literature show that poverty, low educational level, stressful life events (such as divorce or death of a spouse), and obesity can increase the risk of severe headache or migraine (28–31). Education, PIR, marriage, and BMI are potential confounding factors that may affect the CDAI and the risk for migraine. Smoking can cause oxidative stress and increase the risk of vascular events. Furthermore, smokers are also more likely than non-smokers to have severe headaches or migraines (32, 33). Therefore, Model 3 adjusted for PIR, education, marriage, BMI, and smoking.

Previous cross-sectional surveys have revealed that a high CDAI is associated with a low risk of diabetes, hypertension, coronary heart disease, and stroke (15, 34–36), whereas chronic disease status is closely related to the occurrence and development of headache. Liu Wang et al. (37) found that blood pressure is negatively correlated with the occurrence of headache. A prospective population-based study identified blood pressure as a risk factor for headache and migraine (38). Rivera-Mancilla et al. (39) showed that people who are obese and those with diabetes were more likely to experience migraines. Several studies have reported an association of severe headaches or migraines with coronary heart disease and stroke (40–42). Other studies have found an increase in the prevalence and incidence of migraines in patients with asthma (43). A study by Tana et al. (44) reported a correlation between the frequency and pain of migraine and total cholesterol, which was clearly positive. A population-based follow-up study showed that levels of C-reactive protein (CRP) were related to an increased risk of migraine (45). A review of CRP and migraine showed significantly higher CRP concentrations in patients with migra**i**n**e** than in the controls (46). Therefore, in Model 3, we adjusted for five common diseases (hypertension, diabetes, stroke, coronary heart disease, and asthma) and two serological indicators (total cholesterol and CRP).

Studies by Razeghi Jahromi et al. (47) suggest that high-protein, low-carb, and low-calorie diets are effective strategies for preventing headaches and migraines. A randomized controlled trial showed that a very low-calorie ketogenic diet was more effective than a low-calorie balanced diet in preventing high-frequency migraine attacks (48). Therefore, in the multivariate regression analysis, protein, carbohydrate, and capacity intake were also adjusted as potential confounders.

We then adjusted for all potential confounding factors and used restricted cubic spline regression analyses to illustrate the curvy relationship between the CDAI and severe headache or migraine. The association of CDAI with severe headache or migraine was further explored with participants stratified by sex (male/female), age (20–40/40–60 years old), PIR (<2.0/2.0–4.0/≥4.0), education (less than high school/high school or equivalent/more than high school), and BMI (<25.00/25.00–30.00/≥ 30.00).

## 3 Results

### 3.1 Baseline characteristics

Table 1 shows the baseline characteristics of all participants by CDAI quartile. Sex, age, marital status, educational level, PIR, smoking, hypertension, severe headache or migraine, energy, protein, carbohydrate, vitamin A, vitamin C, vitamin E, carotenoid, zinc, selenium and CRP in the different quartiles were found to have statistically significant differences (*p* < 0.05). The mean age of the participants was 39.6 ± 11.5 years, including 2,421 females (49.0%) and 2,522 males (51.0%). Compared to the first quartile array, the group with the high CDAI tended to be younger, have a higher proportion of men, be married, cohabitate or unmarried, have a higher level of education, a higher economic status and a lower CRP. Participants who had never smoked cigarettes had higher CDAIs. Compared to the first quartile array, participants in the higher CDAI group were relatively less likely to have hypertension, severe headache or migraine. Participants with higher CDAIs had higher intakes of energy, carbohydrates, protein, vitamin A, vitamin C, vitamin E, zinc, selenium, and carotenoids.

**Table 1:** Demographic characteristics stratified by CDAI quartiles (*N* = 4,943).

Variable	Total (*N* = 4,943)	Quartile1 (−6.77,−2.63)	Quartile2 (−2.62,−0.76)	Quartile3 (−0.75,1.65)	Quartile 4 (1.66,31.39)	*p*
N	4,943	1,236	1,235	1,237	1,235	
Age (years)	39.6 ± 11.5	40.3 ± 11.8	39.8 ± 11.3	39.5 ± 11.6	38.7 ± 11.1	0.007
Sex, *n* (%)						<0.001
Male	2,522 (51.0)	432 (35)	543 (44)	683 (55.2)	864 (70)	
Female	2,421 (49.0)	804 (65)	692 (56)	554 (44.8)	371 (30)	
Marital status, *n* (%)						<0.001
Married/living with partner	3,189 (64.5)	753 (60.9)	815 (66)	804 (65)	817 (66.2)	
Divorced/separated/widowed	721 (14.6)	222 (18)	180 (14.6)	176 (14.2)	143 (11.6)	
Never married	1,033 (20.9)	261 (21.1)	240 (19.4)	257 (20.8)	275 (22.3)	
Educational level, *n* (%)						<0.001
Less than high school	1,152 (23.3)	354 (28.6)	273 (22.1)	277 (22.4)	248 (20.1)	
High school or equivalent	1,207 (24.4)	307 (24.8)	334 (27)	283 (22.9)	283 (22.9)	
More than high school	2,584 (52.3)	575 (46.5)	628 (50.9)	677 (54.7)	704 (57)	
Smoke status, *n* (%)						<0.001
Current	1,442 (29.2)	422 (34.1)	368 (29.8)	348 (28.1)	304 (24.6)	
Former	961 (19.4)	203 (16.4)	241 (19.5)	264 (21.3)	253 (20.5)	
Never	2,540 (51.4)	611 (49.4)	626 (50.7)	625 (50.5)	678 (54.9)	
PIR	2.6 (1.3, 4.5)	2.1 (1.1, 4.0)	2.5 (1.3, 4.5)	2.8 (1.3, 4.8)	2.7 (1.4, 4.9)	<0.001
BMI (kg/m^2^)	28.4 ± 6.5	28.7 ± 6.9	28.4 ± 6.4	28.3 ± 6.5	28.2 ± 6.3	0.424
Hypertension, *n* (%)	1,088 (22.0)	322 (26.1)	253 (20.5)	263 (21.3)	250 (20.2)	0.001
Diabetes, *n* (%)						0.501
Yes	287 (5.8)	86 (7)	68 (5.5)	68 (5.5)	65 (5.3)	
Borderline	49 (1.0)	13 (1.1)	14 (1.1)	13 (1.1)	9 (0.7)	
Asthma, *n* (%)	595 (12.0)	154 (12.5)	145 (11.7)	144 (11.6)	152 (12.3)	0.901
Coronary heart disease, *n* (%)	86 (1.7)	31 (2.5)	19 (1.5)	20 (1.6)	16 (1.3)	0.107
Stroke, *n* (%)	61 (1.2)	17 (1.4)	14 (1.1)	18 (1.5)	12 (1)	0.683
Severe headache or migraine, *n* (%)	1,232 (24.9)	374 (30.3)	314 (25.4)	282 (22.8)	262 (21.2)	<0.001
Energy (kcal/day)	2351.7 ± 1112.1	1447.5 ± 567.1	2053.9 ± 631.4	2523.3 ± 765.5	3382.5 ± 1282.7	<0.001
Protein intake (g/day)	79.3 (56.5, 107.8)	46.9 (34.2, 61.1)	73.3 (58.7, 87.6)	91.0 (72.9, 113.4)	122.4 (95.4, 158.4)	<0.001
Carbohydrate intake (g/day)	264.3 (186.1, 359.5)	175.3 (125.6, 228.6)	242.6 (184.7, 311.7)	294.0 (226.8, 368.4)	378.3 (294.1, 481.3)	<0.001
Total cholesterol (mg/dl)	199.8 ± 43.5	201.0 ± 45.2	198.8 ± 39.3	201.0 ± 43.4	198.5 ± 45.7	0.306
C-reactive protein (mg/dl)	0.2 (0.1, 0.4)	0.2 (0.1, 0.6)	0.2 (0.1, 0.5)	0.2 (0.1, 0.4)	0.1 (0.1, 0.3)	<0.001
Vitamin E intake (mg/day)	5.9 (3.8, 9.0)	3.0 (2.0, 4.1)	5.1 (3.9, 6.5)	6.9 (5.3, 8.8)	11.0 (8.5, 15.0)	<0.001
Vitamin A intake (mcg/day)	430.0 (228.0, 744.5)	180.0 (92.8, 311.0)	365.0 (228.5, 548.5)	547.0 (346.0, 813.0)	853.0 (556.0, 1278.0)	<0.001
Vitamin C intake (mg/day)	55.3 (22.6, 127.8)	18.6 (8.4, 36.9)	42.3 (22.4, 88.9)	72.7 (36.5, 147.6)	136.3 (68.7, 254.3)	<0.001
Zinc intake (mg/day)	10.7 (7.1, 15.7)	5.9 (4.2, 7.8)	9.6 (7.4, 12.2)	12.8 (9.7, 16.4)	17.7 (13.3, 24.0)	<0.001
Selenium intake (mcg/day)	100.7 (70.4, 141.6)	60.7 (42.2, 79.1)	92.8 (73.2, 114.4)	117.2 (92.5, 145.0)	166.1 (125.7, 221.0)	<0.001
Carotenoid intake (mcg/day)	5731.0 (1946.5, 13394.5)	1552.5 (502.5, 3991.2)	4713.0 (2082.5, 9175.0)	8210.0 (3610.0, 14570.0)	16561.0 (7540.0, 31664.5)	<0.001

CDAI, composite dietary antioxidant index; PIR, poverty income ratio; BMI, body mass index.

### 3.2 Univariate analysis

Table 2 presents the results of the univariate analysis. Statistically significant associations of age, sex, educational level, marital status, PIR, smoking status, and BMI with severe headache or migraine were found. Women were more likely than men to experience severe headaches or migraines within 3 months, with an odds ratio (OR) and a 95% Confidence Interval (CI) of 2.31 (2.02–2.64, *p* < 0.001). Participants who were divorced/separated/widowed, had less than a high school education, smoked cigarettes, had high blood pressure, diabetes, asthma, stroke, or a high BMI were more likely to experience severe headaches or migraines.

**Table 2:** Associations of covariates and the risk of severe headache or migraine.

Variable	OR (95% CI)	*p*	Variable	OR (95% CI)	*p*
Age (years)	0.99 (0.99–1)	0.002	Carbohydrate intake (g/day)	1.00 (1.00–1.00)	0.068
Sex, *n*(%)			Hypertension, *n*(%)		
Male	1 (Reference)		Yes	1 (Reference)	
Female	2.31 (2.02–2.64)	<0.001	No	0.75 (0.64–0.87)	<0.001
Marital status, *n* (%)			Diabetes, n (%)		
Married/Living with partner	1 (Reference)		Yes	1 (Reference)	
Divorced/separated/widowed	1.3 (1.09–1.56)	0.004	No	0.73 (0.57–0.95)	0.02
Never married	1.01 (0.86–1.19)	0.921	Borderline	0.9 (0.46–1.77)	0.769
Educational level, *n*(%)			Asthma, n (%)		
Less than high school	1 (Reference)		Yes	1 (Reference)	
High school or equivalent	0.82 (0.68–0.98)	0.03	No	0.6 (0.5–0.72)	<0.001
More than high school	0.71 (0.61–0.83)	<0.001	Coronary heart disease, n (%)		
Smoking status, *n*(%)			Yes	1 (Reference)	
Current	1 (Reference)		No	0.97 (0.59–1.57)	0.887
Former	0.65 (0.54–0.79)	<0.001	Stroke, n (%)		
Never	0.82 (0.7–0.94)	0.006	Yes	1 (Reference)	
PIR	0.82 (0.79–0.85)	<0.001	No	0.41 (0.25–0.69)	0.001
BMI	1.02 (1.01–1.02)	0.002	C-reactive protein	1.06 (0.99–1.14)	0.113
Energy (kcal/day)	1.00 (1.00–1.00)	<0.001	Total cholesterol	1.00 (1.00–1.00)	0.083
Protein intake (g/day)	1.00(1.00–1.00)	<0.001	CDAI	0.96 (0.94–0.98)	<0.001

OR, odds ratio; 95% CI, 95% confidence interval; CDAI, composite dietary antioxidant index; PIR, poverty income ratio; BMI, body mass index.

### 3.3 Association between the CDAI and severe headache or migraines

**Figure 2:**
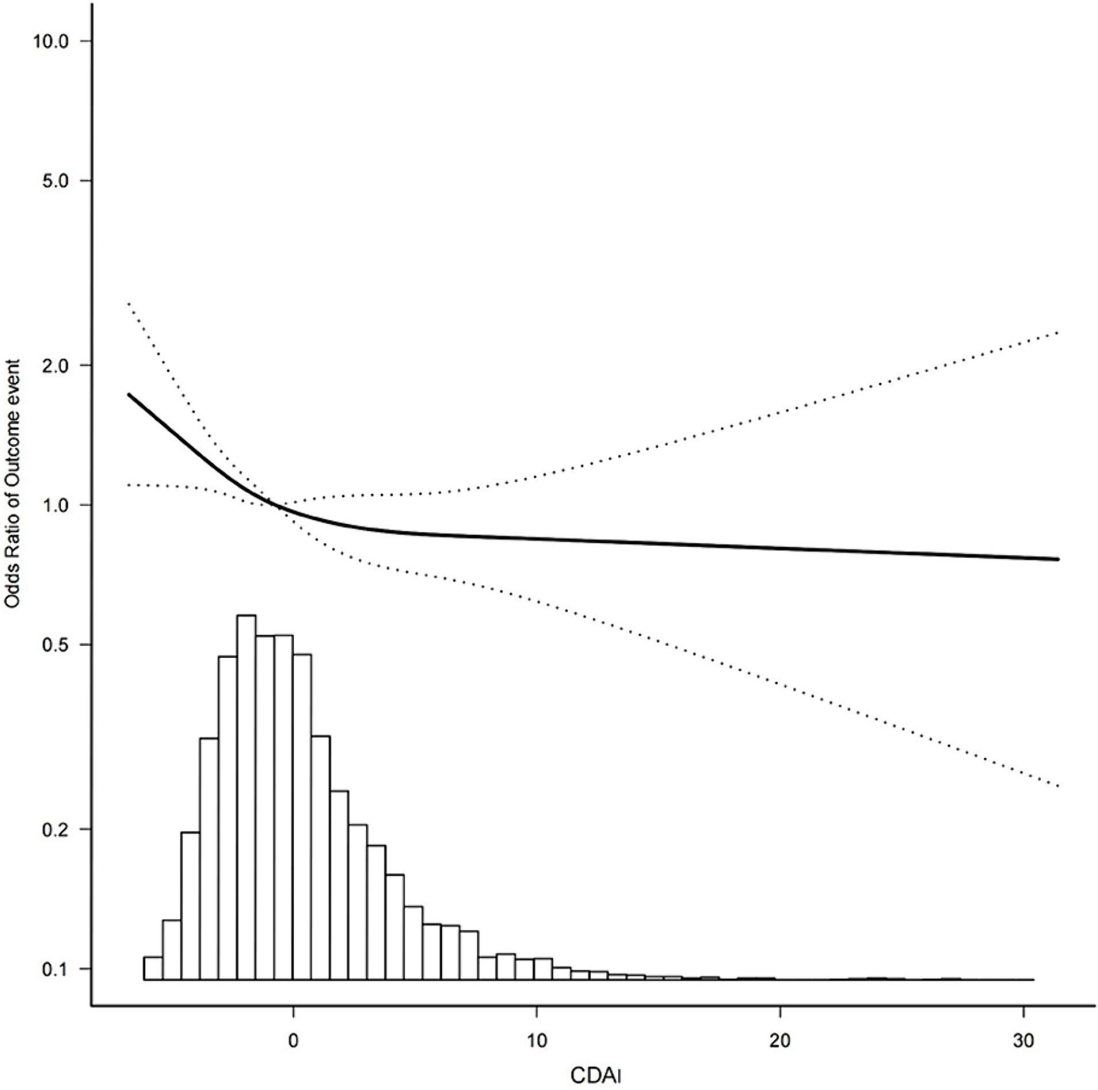
Association between the CDAI and the outcome event’s odds ratio. Outcome event: severe headache or migraine; CDAI, composite dietary antioxidant index.

Table 3 presents the results of the multivariate analysis, in which the relationship between the CDAI and headache were analyzed further. In this analysis, we constructed three models to investigate the relationship between the CDAI and severe headache or migraine. Model 1 was not adjusted. Model 2 was adjusted for age and sex. Model 3 was adjusted for age, sex, marital status, PIR, education, smoking, BMI, hypertension, diabetes, asthma, coronary heart disease, stroke, carbohydrate intake, energy, protein intake, CRP, and total cholesterol. The OR for Model 1 was 0.96 (95% CI = 0.94–0.98, *p* = 0.001), suggesting that the CDAI (continuous value) was negatively associated with severe headache or migraine. After correcting for all of the covariates in Model 3, the OR was found to be 0.97 (95% CI = 0.95–1.00, *p* = 0.048), indicating that for each unit increase in the CDAI, the risk of severe headache or migraine was reduced by 3%. We then grouped the CDAI quartiles to explore the relationship between the CDAI and outcome events. After adjusting for potential confounders, compared with individuals with low CDAIs in the Q1 group, the adjusted ORs between the CDAI and severe headache or migraine in the Q2, Q3, and Q4 groups were 0.84 (95% Cl, 0.69–1.01, *p* = 0.07), 0.77 (95% Cl, 0.63–0.96, *p* = 0.017), and 0.73 (95% Cl, 0.56–0.95, *p* = 0.02), respectively. Compared to patients in Q1, the patients in Q3 and Q4 had a 23 and 27% reduction in migraine prevalence, respectively. Moreover, the *p*-value for trend was significant (*p* = 0.014). We also analyzed the data to determine whether there was a nonlinear correlation between the CDAI and headache. After adjusting for age, sex, education, marital status, PIR, smoking, BMI, hypertension, diabetes, asthma, coronary heart disease, stroke, energy, protein, carbohydrates, CRP, and total cholesterol, the CDAI showed an L-shaped relationship with severe headache or migraine (Figure 2). The results of RCS analysis showed that when CDAI was less than about 2.0, the odds ratio of severe headache or migraine decreased significantly with the increase of CDAI. When CDAI is greater than about 2.0, the odds ratio of severe headache or migraine does not decrease significantly.

**Table 3:** Association of CDAI, CDAI quartile and severe headache or migraine base on logistic regression analysis.

	Model 1		Model 2		Model 3	
	OR (95% CI)	*p*	OR (95% CI)	*p*	OR (95% CI)	*p*
CDAI	0.96 (0.94–0.98)	<0.001	0.98 (0.97–1.00)	0.104	0.97 (0.95–1.00)	0.048
**Categories**						
Q1	1(Ref)		1(Ref)		1(Ref)	
Q2	0.79 (0.66–0.94)	0.007	0.83 (0.7–1.00)	0.047	0.84 (0.69–1.01)	0.07
Q3	0.68 (0.57–0.81)	<0.001	0.78 (0.65–0.94)	0.01	0.77 (0.63–0.96)	0.017
Q4	0.62 (0.52–0.74)	<0.001	0.8 (0.66–0.97)	0.024	0.73 (0.56–0.95)	0.02
Trend.test	<0.001		0.015		0.014	

Three models were used to observe the association between CDAI and severe headache or migraine. Model 1 was not adjusted. Model 2 was adjusted for age and sex. Model 3 was adjusted for age, sex, education, marital status, PIR, smoking, BMI, hypertension, diabetes, asthma, coronary heart disease, stroke, energy, protein intake, carbohydrate intake, C-reactive protein, and total cholesterol. CDAI, composite dietary antioxidant index; NHANES, National Health and Nutrition Examination Survey; OR, odds ratio; 95% CI, 95% confidence interval; Q, Quartile.

### 3.4 Subgroup analysis of CDAI and severe headache or migraine

**Figure 3:**
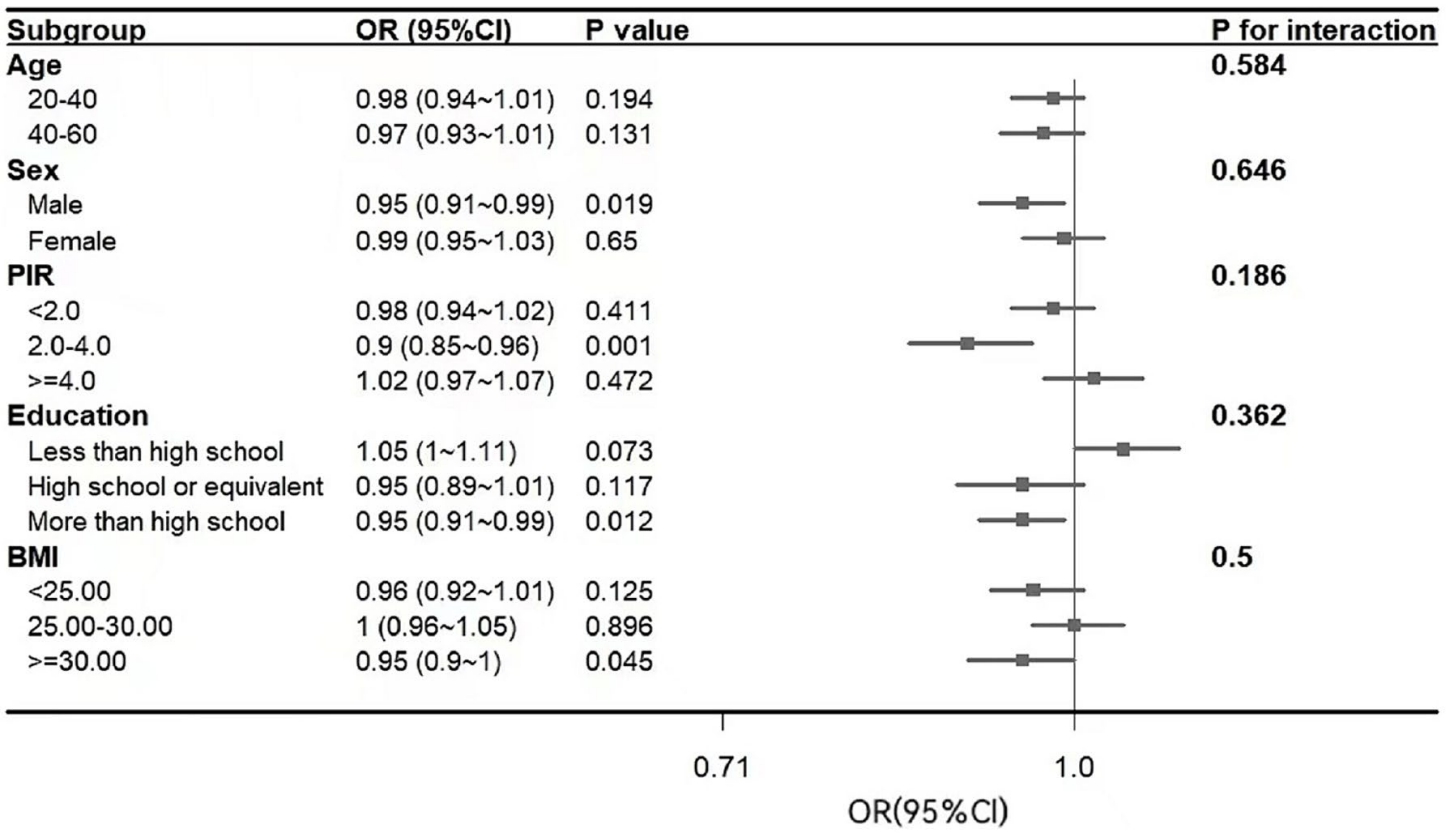
Subgroup analysis of the relationship of the CDAI with severe headache or migraine. In addition to stratifying the variables, each stratification was adjusted for all other variables. CDAI, composite dietary antioxidant index; OR, odds ratio; CI, confidence interval.

Subgroup analysis was conducted to explore potential associations between the CDAI and severe headache or migraine among the different study groups based on their age, sex, socio-economic level, educational level, and BMI (Figure 3). The study revealed that in some subgroups, higher CDAI was not always associated with a lower risk of migraine. Subgroup analysis showed that this association was not statistically significant among age subgroups, low income, high income, education below high school, and non-obese groups (*p* > 0.05). The results revealed that CDAI was negatively associated with severe headache or migraine in males, those with a high educational level, 2 < PIR < 4, and a BMI ≥ 30.00 (*p* < 0.05). No significant interactions were observed for all subgroup variables (all *p*-values for interaction >0.05).

## 4 Discussion

There was a negative association between CDAI and migraine prevalence, which also remained stable after adjusting for potential confounders. Participants with the highest quartile of CDAI were 27 percent less likely to experience severe headache or migraine compared to those with the lowest quartile.

Oxidative stress refers to a state of imbalance between oxidation and antioxidant action in the body, which tends to oxidize. In response to nutritional imbalance, infection, drugs, environmental pollution, and other factors, the human body produces excessive free radicals, which causes damage to the intracellular antioxidant defense system. This process results in lipid peroxidation and degeneration of biological macromolecules, such as proteins and nucleic acids, leading to various adverse consequences. Current medical research has reported that oxidative stress damage promotes the occurrence and development of neurodegenerative diseases, tumors, cardiovascular diseases, osteoarthritis, and other diseases. Most severe headaches and migraines are categorized as neurovascular diseases, and there is extensive evidence showing that oxidative stress is one of the factors in the pathogenesis of severe headaches or migraines, which suggests that the use of antioxidants is an effective strategy for the prevention and cure of severe headaches or migraines. It seems that higher dietary antioxidant intake or antioxidant supplement intake can regulate headache symptoms in patients. Several studies have reported a relationship between dietary antioxidants and headaches. For example, Liu et al. (49) found that niacin intake in the diet of American adults showed an L-shaped relationship with migraines, and the inflection point was about 21.0 mg/day. Zheng Heqing et al. used the NHANES data to conduct a cross-sectional study that revealed a salient association between a higher zinc intake and a lower incidence of migraine (22, 50). A randomized controlled trial on migraine found that treatment with a proprietary supplement containing magnesium, riboflavin, and coenzyme Q10 could effectively reduce the frequency of migraines and relieve pain (51). Another study showed that 95 patients with migraine who supplemented their diets with folic acid and pyridoxine for 3 months significantly reduced the severity of migraine as well as the frequency and duration of headaches (52). These studies further support the protective effect of antioxidants against severe headache or migraine.

However, the effect of diet on the body’s oxidative environment is often a synergistic effect of multiple antioxidant components, and considering the mixed composition of nutrients in foods, evaluating the overall dietary antioxidant intake could provide information that is more comprehensive. The CDAI is a measure of the total antioxidant level in the diet that reflects the antioxidant effect of the overall diet. The existing literature shows that a high CDAI can eliminate inflammatory factors and reduce the risk of a variety of common diseases, such as lung cancer, hypertension, chronic kidney disease, heart failure, aging, and depression. Existing studies (11, 20, 21) also show that the CDAI is an evaluation tool worthy of further examination; however, the relationship between the CDAI and severe headache or migraine has not been reported. The purpose of this study was to fill the research gap and conduct a comprehensive assessment of the effects of various antioxidants on the occurrence of severe headache or migraine. In this cross-sectional study, which analyzed data from the NHANES, we found that CDAI was inversely associated with severe headache or migraine. Previous studies have shown that subjects with lower total dietary antioxidant capacity scores had a significantly higher frequency of migraine attacks, and the present study further supports this conclusion (53). After adjusting for all the confounding factors, the limited cubic spline showed an L-shaped curve between the CDAI and severe headache or migraine. As illustrated in Figure 2, the odds ratio of severe headache or migraine decreases as CDAIs increase, and we see a saturation effect, which suggests that the likelihood of experiencing severe headache or migraine is relatively low at high CDAI. In the subgroup analysis, the negative association between the CDAI and headache seem to be stronger among males and obese, middle-income, and higher-educational groups. But the interactions were not statistically significant (p for interaction>0.05). Subgroup analysis was stratified by age, sex, socio-economic level, educational level, and BMI, and no significant interaction was observed for all subgroup variables. This indicates that the association between CDAI and severe headache or migraine may not be affected by these factors. However, the results of subgroup analysis are only preliminary exploration, and more relevant studies are needed to further validate.

The occurrence of migraine is influenced by many factors, including genetic component, environmental factors, diet, metabolism, etc. In this cross-sectional study, although the negative association between CDAI and migraine remained stable after adjusting for multiple confounders, there are still potential factors such as genetic component that may effect this association. The frequency and severity of migraine are also affected by a variety of factors, including diet, sleep, fatigue, environment, etc. However, because the NHANES database does not provide data on the frequency and severity of severe headache or migraine, we were unable to further assess the relationship between CDAI and characteristics of severe headache or migraine (including frequency, duration, and severity), which is one of the limitations of the study. Although there is a genetic predisposition to migraine, lifestyle should still be considered in migraine management. According to existing studies, oxidative stress is considered to be a pathological cause of migraine. Previous research (6–8) had found that the levels of plasma markers of oxidative stress such as 4-hydroxy-2-nonenal (HNE) and malondialdehyde (MDA) in migraine patients were significantly increased. Elevated levels of oxidative stress can not only induce migraine, but also aggravate the frequency and pain of migraine (8, 54). At present, there is a lot of research evidence to support that diet is one of the effective factors in regulating oxidative conditions *in vivo*, so finding dietary methods to control oxidative stress is a worthy area of attention. Previous studies have shown that dietary patterns rich in antioxidants can combat inflammation, reduce oxidative stress levels, and improve the frequency, duration, and severity of migraine (55, 56). As a dietary antioxidant score, CDAI represents the antioxidant level of an individual’s diet. Vitamin A, vitamin C, vitamin E, zinc, selenium are often considered to be the key components of CDAI, which have the ability to neutralize free radicals, anti-inflammatory, and antioxidant damage (57–60). Existing literature has shown that high CDAI can eliminate inflammatory factors and reduce the risk of various cancers (11–13), hypertension (15), cardiovascular disease (21), depression (20), etc. These studies suggest that high CDAI may reduce the risk of associated diseases by enhancing antioxidant defense mechanisms. A previous study showed that women with high CDAI levels had significantly lower migraine frequency (53). Based on the characteristics of CDAI and the clinical characteristics of migraine, we speculate that the influence of CDAI on migraine may be multifaceted, which may have an impact on the occurrence, frequency and degree of migraine.

The present study suggests that a higher CDAI is a protective factor for severe headache or migraine. The findings of this study also provide useful information about nutritional supplements, which may serve as a guide. The synergistic effect of various antioxidant components maintains the balance of the oxidative environment in humans. Many clinical trials have confirmed that a combination of various antioxidant components is beneficial and safe for preventing migraine in adult patients. For example, a randomized controlled trial revealed that in contrast to the placebo group, people using proprietary supplements containing riboflavin, magnesium, and Q10 had significantly reduced headache severity and duration (48). A prospective observational study demonstrated that the combination of coenzyme Q10, feverfew, and magnesium could significantly reduce the number of headache days experienced by individuals and improve their quality of life (61). A clinical study of Greek patients with migraines found that a fixed combination supplement of magnesium, vitamin B2, feverfew, *Andrographis paniculata*, and coenzyme Q10 appeared to be an effective and well-tolerated way to prevent migraine (62). These results suggest that it is feasible and necessary to combine multiple antioxidant components in the prevention and treatment of headache. Based on our results and those of other studies, we believe that more studies are worth conducting to guide the manufacturing of proprietary supplements containing various antioxidants to combat oxidative stress effectively and prevent severe headaches or migraines.

This study has the following limitations: (1) Due to the study’s long period of data collection and the complicated method of constructing CDAIs, there may be measurement errors and inaccuracies in the dietary assessments; (2) The population observed in this study was American (US) adults aged 20–60 years, and whether the results can be generalized to other populations remains to be verified; (3) Despite the adjustments made for potential confounders, residual confounders that may still be present could affect the association of the CDAI with severe headache or migraine. (4) Due to the lack of data on the frequency, severity, and duration of severe headache or migraine in NHANES database, our study was unable to further assess the relationship between CDAI and specific features of severe headache or migraine. (5) The study relied on self-reported results from the NHANES pain questionnaire and results from the American Migraine Prevalence and Prevention study to define migraine, an approach that could not be cross-validated against the International Classification of Headache Disorders (ICHD) diagnostic criteria, raising concerns about the accuracy of migraine diagnosis. This approach has been used in previous studies to assess migraine. And we think study results can provide some meaningful insights for future research. Of course, future studies will need to use the ICHD 3rd Edition migraine diagnostic criteria to further confirm our results. (6) The current study is a cross-sectional study that only suggests an association between CDAI and severe headache or migraine, and a causal relationship between CDAI and severe headache or migraine cannot be established.

## 5 Conclusion

In summary, our study found that higher CDAI was associated with a reduced prevalence of severe headache or migraine. More randomized controlled trials or cohort studies are warranted to further validate the finding.

## Data availability statement

The original contributions presented in the study are included in the article/supplementary material, further inquiries can be directed to the corresponding author.

## Ethics statement

Ethical approval was not required for the study involving humans in accordance with the local legislation and institutional requirements. Written informed consent to participate in this study was not required from the participants or the participants’ legal guardians/next of kin in accordance with the national legislation and the institutional requirements.

## Author contributions

ZZ: Conceptualization, Data curation, Formal analysis, Methodology, Writing – original draft, Writing – review & editing. XC: Conceptualization, Formal analysis, Methodology, Validation, Writing – original draft. HF: Data curation, Formal analysis, Methodology, Validation, Writing – original draft. JY: Data curation, Formal analysis, Methodology, Writing – original draft. XT: Conceptualization, Funding acquisition, Project administration, Supervision, Writing – original draft. RH: Conceptualization, Funding acquisition, Project administration, Supervision, Writing – original draft, Writing – review & editing.

## Footnotes

^1^ https://www.cdc.gov/nchs/nhanes/
